# Serum alpha-1 acid glycoprotein and gallstone risk in US adult women: a cross-sectional analysis of the NHANES

**DOI:** 10.3389/fnut.2025.1527717

**Published:** 2025-02-12

**Authors:** Xiaocheng Li, Peiling He

**Affiliations:** ^1^Department of Hepatopancreatobiliary Surgery, Third Affiliated Hospital of Zunyi Medical University (The First People’s Hospital of Zunyi), Zunyi, China; ^2^Department of Medical Equipment, Third Affiliated Hospital of Zunyi Medical University (The First People’s Hospital of Zunyi), Zunyi, China

**Keywords:** alpha-1 acid glycoprotein, gallstone, cross-sectional analysis, NHANES, inflammation

## Abstract

**Background:**

Gallstone disease, a common biliary disorder, is linked to inflammation and immune responses. However, the association between serum alpha-1 acid glycoprotein (AGP), a key inflammatory marker, and gallstone risk remains underexplored.

**Methods:**

Data from the National Health and Nutrition Examination Survey (NHANES) 2017–2020 and 2021–2023 cycles were analyzed. Gallstone disease was determined by self-reported physician diagnosis. Serum AGP levels were measured using a high-sensitivity turbidimetric immunoassay. Weighted logistic regression, subgroup analyses, smoothed curve analysis, and multiple imputation were used to examine the relationship between AGP and gallstone risk.

**Results:**

This cross-sectional analysis included 1,903 adult women in the U.S. aged 20–49. After adjusting for all covariates, serum AGP levels were positively associated with gallstone risk (OR: 3.07; 95% CI: 1.16, 8.11; *p* = 0.036). Compared to the first tertile (T1), the third AGP tertile (T3) had an OR of 1.87 (95% CI: 1.11, 3.14; *p* = 0.030). Smoothed curve analysis indicated a positive relationship between AGP and gallstone risk. Subgroup analyses consistently demonstrated this positive association across various demographic and clinical categories, with significant interactions observed for the ratio of family income to poverty. Sensitivity analyses using multiple imputation further supported the conclusion that AGP was associated with increased gallstone risk.

**Conclusion:**

AGP is significantly associated with an increased risk of gallstones in U.S. adult women, suggesting its potential as a biomarker for risk stratification. Further research is needed to elucidate the underlying mechanisms and potential causal relationships.

## 1 Introduction

Gallstone disease represents a prevalent disorder of the biliary system, influenced by multiple mechanisms including genetic predisposition, lifestyle factors, and metabolic abnormalities (1–3). Common risk factors for gallstones include being female, older age, type 2 diabetes, non-alcoholic fatty liver disease, obesity, and rapid weight loss (4, 5). Gallstones are typically classified as either cholesterol or pigment stones, with cholesterol stones being the most common. Although the exact mechanism of gallstone formation remains unclear, it is thought to primarily involve genetic predisposition, cholesterol crystallization, and bile supersaturation (6). Recent research has increasingly focused on the roles of inflammation and the immune system in the pathogenesis of gallstones (7, 8). Serum biomarkers of inflammation and immune modulation have emerged as critical indicators in this area of study, garnering significant attention.

Alpha-1 acid glycoprotein (AGP), also known as orosomucoid, is a heavily glycosylated glycoprotein that plays a pivotal role in inflammation and immune responses (9, 10). Its expression levels significantly increase during both acute and chronic inflammatory processes, such as those observed in cancer, obesity, infection, and cardiovascular disease, reflecting alterations in the body’s inflammatory status and immune activity. Metabolic conditions, characterized by chronic systemic inflammation (11), are believed to play a central role in gallstone formation by disrupting bile acid homeostasis, increasing cholesterol saturation in bile, and impairing gallbladder motility (12). A study has suggested that AGP may have an impact on bile acid metabolism (13). Therefore, there appears to be a potential association between AGP and gallstones, which may be mediated by its impact on bile acid metabolism and chronic inflammation induced by metabolic dysregulation. However, research on the relationship between AGP and gallstones is limited and somewhat inconsistent. A case–control study has suggested a positive correlation between serum AGP levels and gallstones (14). In contrast, another study involving bile samples from 12 gallstone patients has found no effect of AGP on concanavalin A activity, raising questions about AGP’s role in gallstone formation (15). Thus, elucidating the relationship between AGP and gallstones remains of significant importance.

This study will investigate the association between AGP and gallstone prevalence in women using data from the National Health and Nutrition Examination Survey (NHANES) 2017–2020 and 2021–2023 cycles. Weighted logistic regression, subgroup analyses, and smoothed curve analysis will be conducted to explore this relationship. Multiple imputation (MI) will also be performed to validate the robustness of the findings.

## 2 Methods

### 2.1 Research design and participants

The National Center for Health Statistics (NCHS) administers the NHANES program, which collects comprehensive health data from a representative sample of adults and children through interviews, physical examinations, and laboratory analyses. The NCHS Institutional Review Board approved the study protocol, and informed consent was obtained from all participants. Detailed information and access to the data are available on the NHANES website.^1^

The study population was limited to females aged 20–49 years for several reasons. On one hand, AGP measurements in the NHANES dataset were only available for females aged 12–49 years and children aged 1–5 years during the 2017–2020 and 2021–2023 cycles, while males were not included in the AGP measurements, representing a limitation of the dataset. On the other hand, the gallstone questionnaire, which was used to identify participants’ gallstone history, was only administered to individuals aged 20 years and older.

**Figure 1:**
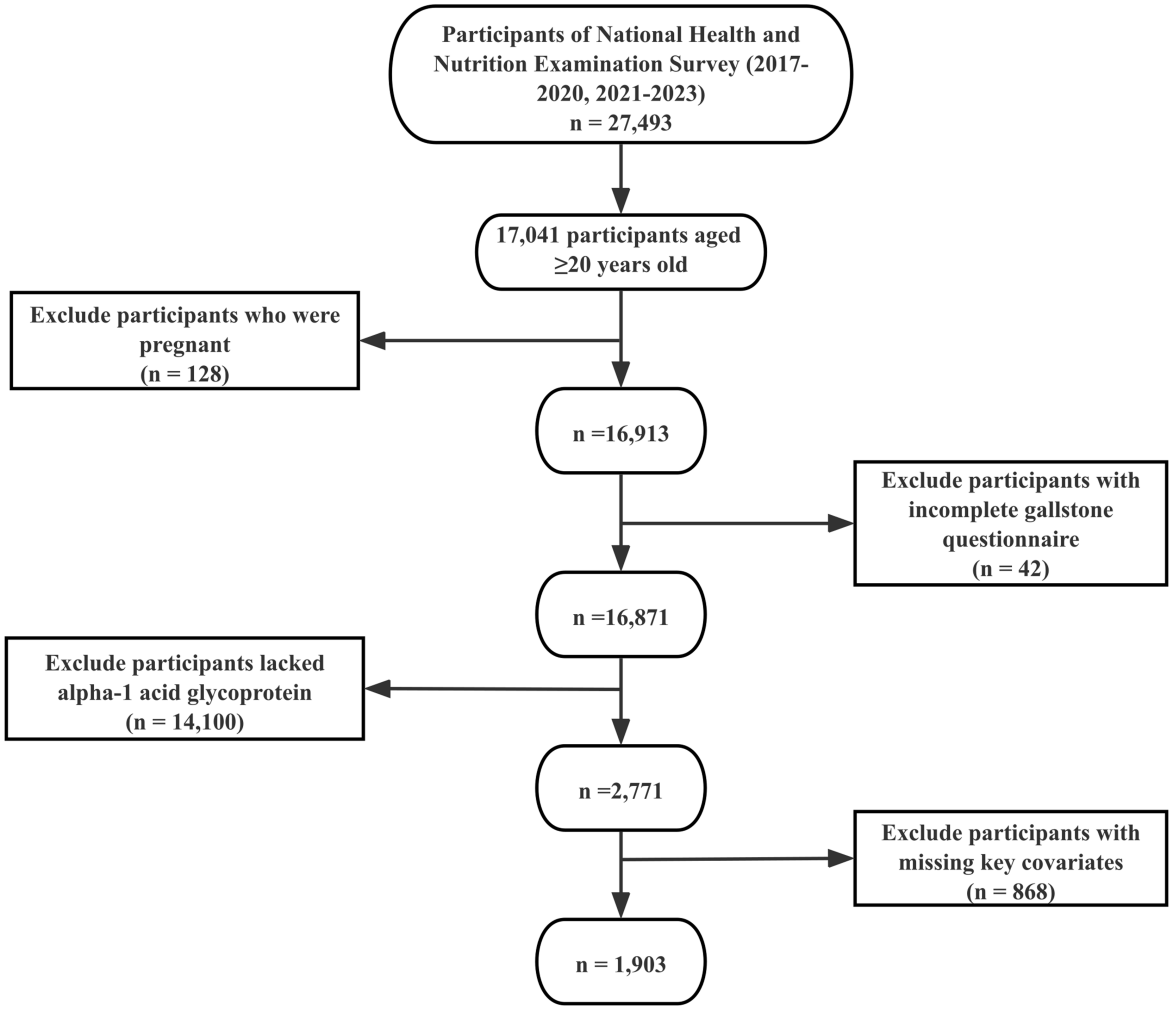
Flowchart of participant selection. The NHANES 2017–2020 and 2021–2023 cycles initially included 27,493 participants. Following the application of inclusion and exclusion criteria, 1,903 participants were ultimately retained for the final analysis.

Of the initial 27,493 enrollees, 10,452 were excluded because they were under 20 years of age and therefore did not complete the gallstone questionnaire. Further exclusions included 128 participants who were pregnant, 42 participants who either refused to answer or were unsure about their gallstone history, 14,100 who lacked serum AGP test results, and 868 participants with incomplete data on key covariates. After these exclusions, the final analytic sample comprised 1,903 participants. The selection process is illustrated in Figure 1, and Supplementary Table 1 provides details on missing data for key covariates.

### 2.2 Definition of gallstone

Participants were assessed for the presence of gallstones by asking, “Has a doctor ever told you that you have gallstones?” Individuals who answered “Yes” were categorized as having gallstones, while those who answered “No” were categorized as not having gallstones.

### 2.3 Measurement of AGP

AGP levels were measured in serum samples as part of the NHANES 2017–2020 and 2021–2023 cycles using a high-sensitivity turbidimetric immunoassay. This method involves the reaction of AGP in the sample with specific antibodies, leading to the formation of antigen–antibody complexes that cause turbidity, which is then quantitatively measured. The assay was performed using a clinical chemistry analyzer, and results were standardized against known controls to ensure accuracy. This robust method allows for the precise quantification of AGP, which is crucial for assessing its association with various health outcomes, including gallstone formation.

### 2.4 Identification of covariates

The statistical model incorporated the following variables as covariates based on previous research (16–18): age (years), race/ethnicity (Non-Hispanic White, Non-Hispanic Black, Other), level of education (less than high school, high school, college or above), ratio of family income to poverty (<1.3, 1.3–3.5, and >3.5), obesity, alcohol intake, sedentary activity, total cholesterol, hs-CRP, total energy intake, total fiber intake, total fat intake, total cholesterol intake, total moisture intake, and history of smoking, diabetes, and hypertension.

Age was divided into two categories: 20–39 years and 40–49 years.

Diabetes was identified based on an HbA1c level of ≥6.5%, a self-reported diabetes diagnosis, a history of using antidiabetic medications, or fasting blood glucose levels of ≥7 mmol/L.

Hypertension was defined by a mean systolic blood pressure of ≥140 mmHg, a mean diastolic blood pressure of ≥90 mmHg, or a self-reported diagnosis of hypertension.

Obesity was determined as a body mass index (BMI) of ≥30 kg/m^2^.

Alcohol intake was assessed using responses to two specific questionnaire items: ALQ121, which asked, “In the past 12 months, how often did you drink alcoholic beverages?” and ALQ131, which queried, “During the past 12 months, on days when you consumed alcoholic beverages, how many drinks did you typically have?” Participants who reported consuming 12 or more alcoholic drinks per year were classified as alcohol consumers.

Individuals classified as smokers if they had smoked at least 100 cigarettes in their lifetime.

Total cholesterol levels and hs-CRP were derived from laboratory test results.

Sedentary activity was assessed using the NHANES questionnaire, which recorded the self-reported average daily time spent in sedentary behaviors, including activities such as sitting and watching television or using a computer, measured in minutes per day.

Total energy intake, total fiber intake, total fat intake, total cholesterol intake, and total moisture intake were derived from the first 24-h dietary recall interview conducted by NHANES. Participants reported all foods and beverages consumed in the previous 24 h, and these data were subsequently processed to quantify the total intake of each nutrient for the first day.

### 2.5 Statistical methods

Two-sided statistical tests were conducted, with significance set at *p* < 0.05. All analyses were performed using R version 4.4.0. The NHANES official website recommends the use of appropriate sampling weights for statistical analysis and provides detailed guidance on how to apply these weights. In this study, the specific sample weights were used, as AGP data were measured in participants aged 3–5 years and females aged 12–49 years. The “survey” package in R was utilized to account for the NHANES complex sampling design, including weights, strata, and primary sampling units (PSUs), ensuring accurate variance estimation and nationally representative results.

Baseline characteristics were analyzed using the “svyCreateTableOne” function, which automatically applies survey-weighted statistical methods. Unweighted data were generated using the “CreateTableOne” function. Continuous variables are presented as weighted means with standard deviations, while categorical variables are presented as unweighted counts with weighted percentages. All statistical tests, including normality tests and non-parametric tests for continuous variables that did not meet the normality assumption, were performed using survey-weighted methods. An overall summary across all groups was also provided.

In accordance with STROBE guidelines (19), three weighted multivariable regression models were constructed. Model 1 was unadjusted for any covariates. Model 2 was adjusted for age, race/ethnicity, level of education, and the ratio of family income to poverty. Model 3 was adjusted for all potential covariates. Potential collinearity among variables included in the complete model (Model 3) was assessed using generalized variance inflation factors (GVIFs) with adjustments for degrees of freedom. The complete GVIF results for all variables are provided in Supplementary Table 2. While some variables, such as ratio of family income to poverty (2.64), total energy intake (4.36), and total fat intake (4.93), exceeded the commonly accepted threshold of √5 (approximately 2.236), these were not central to the primary research objective. Furthermore, even when total energy intake or total fat intake was excluded from the model, the odds ratio (OR), 95% confidence interval (CI), and *p*-values for the association between AGP and gallstone risk showed only slightly changes. Thus, overall multicollinearity was not considered a significant concern for evaluating the association between AGP and gallstone risk.

Subgroup analyses were performed using weighted multivariate regression analysis for all categorical variables. Interaction terms were added to the models, and the log-likelihood ratio test was used to examine heterogeneity in associations across subgroups. A smoothed curve was generated to identify the dose–response relationship between AGP and gallstone risk using generalized additive models (GAM) via the “mgcv” package in R. GAM was chosen for its ability to model non-linear relationships flexibly using penalized regression splines. Non-linearity was assessed by comparing the Akaike information criterion (AIC) values of the GAM and linear models. Additionally, the smoothed curves were visually inspected for deviations from linearity to further confirm the presence of non-linear relationships.

To mitigate bias due to missing data, MI was performed using the “MICE” package in R software (20). The imputation model included variables listed in Supplementary Table 1, selected based on the variables included in model 3 described earlier. MI was performed using predictive mean matching (PMM) with five imputed datasets (m = 5) and 20 iterations to ensure algorithm convergence. Diagnostics, including density plots and trace plots, were performed to validate the stability and convergence of imputed values. The results from the imputed datasets were combined using Rubin’s rules to account for within- and between-imputation variability.

## 3 Results

### 3.1 Baseline characteristics

Table 1 summarizes the baseline characteristics of the study population across tertiles of AGP, adjusted for survey weights. Among the 1,903 participants, gallstones were identified in 185 individuals, corresponding to a sample-weighted prevalence of 9.9%. The weighted tertile ranges for AGP were T1 [0.261–0.674], T2 [0.674–0.880], and T3 [0.880–2.020]. Significant differences were observed among the weighted AGP tertile groups in terms of education level, ratio of family income to poverty, smoking history, obesity, total cholesterol, total fiber intake, hs-CRP, history of diabetes, history of hypertension, and prevalence of gallstones (*p* < 0.05).

**Table 1:** Demographic and clinical characteristics by AGP tertiles from the National Health and Nutrition Examination Survey (NHANES) 2017–2020.

Characteristics		AGP tertiles			*p*
	Overall	T1	T2	T3	
	*N* = 1903	*n* = 620	*n* = 622	*n* = 661	
Age, years					0.239
20–39	1,242 (67.3)	425 (70.7)	407 (65.2)	410 (66.0)	
40–49	661 (32.7)	195 (29.3)	215 (34.8)	251 (34.0)	
Race/Ethnicity, *n* (%)					0.283
Non-Hispanic White	804 (58.2)	244 (57.5)	246 (56.8)	314 (60.4)	
Non-Hispanic Black	384 (12.8)	101 (11.2)	131 (12.9)	152 (14.5)	
Other	715 (28.9)	275 (31.3)	245 (30.3)	195 (25.1)	
Education, *n* (%)					<0.001
Less than high school	215 (7.7)	59 (6.4)	85 (9.1)	71 (7.5)	
High school	331 (19.9)	75 (13.5)	113 (21.1)	143 (25.1)	
College or above	1,357 (72.5)	486 (80.1)	424 (69.8)	447 (67.4)	
Ratio of family income to poverty, *n* (%)					<0.001
<1.3	586 (23.3)	147 (18.4)	205 (25.1)	234 (26.4)	
1.3–3.5	690 (35.6)	207 (31.9)	221 (33.4)	262 (41.6)	
>3.5	627 (41.1)	266 (49.7)	196 (41.5)	165 (32.0)	
Alcohol intake, *n* (%)					0.279
No	821 (38.5)	235 (35.5)	287 (39.1)	299 (41.1)	
Yes	1,082 (61.5)	385 (64.5)	335 (60.9)	362 (58.9)	
Diabetes, *n* (%)					<0.001
No	1738 (92.8)	599 (97.5)	562 (92.1)	577 (88.9)	
Yes	165 (7.2)	21 (2.5)	60 (7.9)	84 (11.1)	
Hypertension, *n* (%)					<0.001
No	1,542 (83.9)	542 (89.2)	506 (84.6)	494 (77.9)	
Yes	361 (16.1)	78 (10.8)	116 (15.4)	167 (22.1)	
Obesity, *n* (%)					<0.001
No	1,057 (58.9)	529 (85.0)	346 (60.9)	182 (30.7)	
Yes	846 (41.1)	91 (15.0)	276 (39.1)	479 (69.3)	
Smoking, *n* (%)					<0.001
No	1,304 (68.9)	480 (78.0)	434 (67.7)	390 (60.9)	
Yes	599 (31.1)	140 (22.0)	188 (32.3)	271 (39.1)	
Gallstones, *n* (%)					<0.001
No	1718 (90.1)	589 (95.4)	563 (90.1)	566 (84.8)	
Yes	185 (9.9)	31 (4.6)	59 (9.9)	95 (15.2)	
Total cholesterol, mmol/L	4.72 (0.90)	4.59 (0.83)	4.79 (0.92)	4.79 (0.93)	0.003
Sedentary activity, minutes	360.59 (206.01)	356.97 (200.31)	346.06 (201.06)	378.91 (215.35)	0.080
Total energy intake, kcal	1888.72 (798.16)	1920.85 (791.63)	1827.74 (813.78)	1917.90 (786.44)	0.198
Total fiber intake, g	14.82 (10.14)	17.00 (12.49)	14.01 (8.59)	13.44 (8.45)	0.002
Total fat intake, g	79.52 (40.98)	82.19 (41.12)	75.87 (39.94)	80.51 (41.69)	0.114
Total cholesterol intake, g	275.48 (223.16)	280.27 (230.37)	286.98 (228.43)	259.06 (209.36)	0.132
Total moisture intake, g	2843.58 (1320.13)	2919.77 (1270.03)	2802.41 (1351.13)	2808.41 (1336.63)	0.162
Hs-CRP, mg/L	4.30 (7.03)	1.80 (3.83)	3.06 (4.07)	8.07 (9.78)	<0.001
AGP, g/L	0.79 (0.23)	0.55 (0.09)	0.78 (0.06)	1.06 (0.15)	<0.001

Continuous variables were presented as weighted means with standard deviations. Means, percentages, and AGP tertiles were adjusted for the survey weights of the National Health and Nutrition Examination Survey (NHANES). AGP, alpha-1 acid glycoprotein; hs-CRP, high-sensitivity C-reactive protein.

### 3.2 Association between AGP and gallstone risk

The association between AGP and the risk of gallstones was examined using three different models, adjusting for various confounders. The results were presented in Table 2.

**Table 2:** Association between AGP and gallstone risk.

Exposure	Model 1		Model 2		Model 3	
	OR (95% CI)	*p*	OR (95% CI)	*p*	OR (95% CI)	*p*
AGP (continuous)	7.35 (3.78, 14.29)	< 0.001	6.88 (3.52, 13.45)	< 0.001	3.07 (1.16, 8.11)	0.036
Tertiles of AGP						
T1, [0.261–0.674]	Ref.		Ref.		Ref.	
T2, [0.674–0.880]	2.28 (1.30, 4.01)	0.007	2.23 (1.25, 3.97)	0.011	1.53 (0.86, 2.69)	0.162
T3, [0.880–2.020]	3.73 (2.36, 5.87)	< 0.001	3.56 (2.28, 5.57)	< 0.001	1.87 (1.11, 3.14)	0.030

Model 1: Unadjusted. Model 2: Adjusted for age, race/ethnicity, education, ratio of family income to poverty. Model 3: Adjusted for age, race/ethnicity, education, ratio of family income to poverty, alcohol intake, obesity, hypertension, diabetes, smoking, sedentary activity, total cholesterol, hs-CRP, total energy intake, total fiber intake, total fat intake, total cholesterol intake, total moisture intake. All results were adjusted for the survey weights of the National Health and Nutrition Examination Survey (NHANES). AGP, alpha-1 acid glycoprotein; hs-CRP, high-sensitivity C-reactive protein; OR, odds ratio; CI, confidence interval.

After adjusting for all covariates (Model 3), AGP, treated as a continuous variable, was still positively associated with gallstone risk (OR: 3.07; 95% CI: 1.16–8.11; *p* = 0.036). Participants in the highest AGP tertile (T3) remained significantly associated with gallstone risk, with an OR of 1.87 (95% CI: 1.11–3.14; *p* = 0.030), compared to those in the lowest tertile (T1).

**Figure 2:**
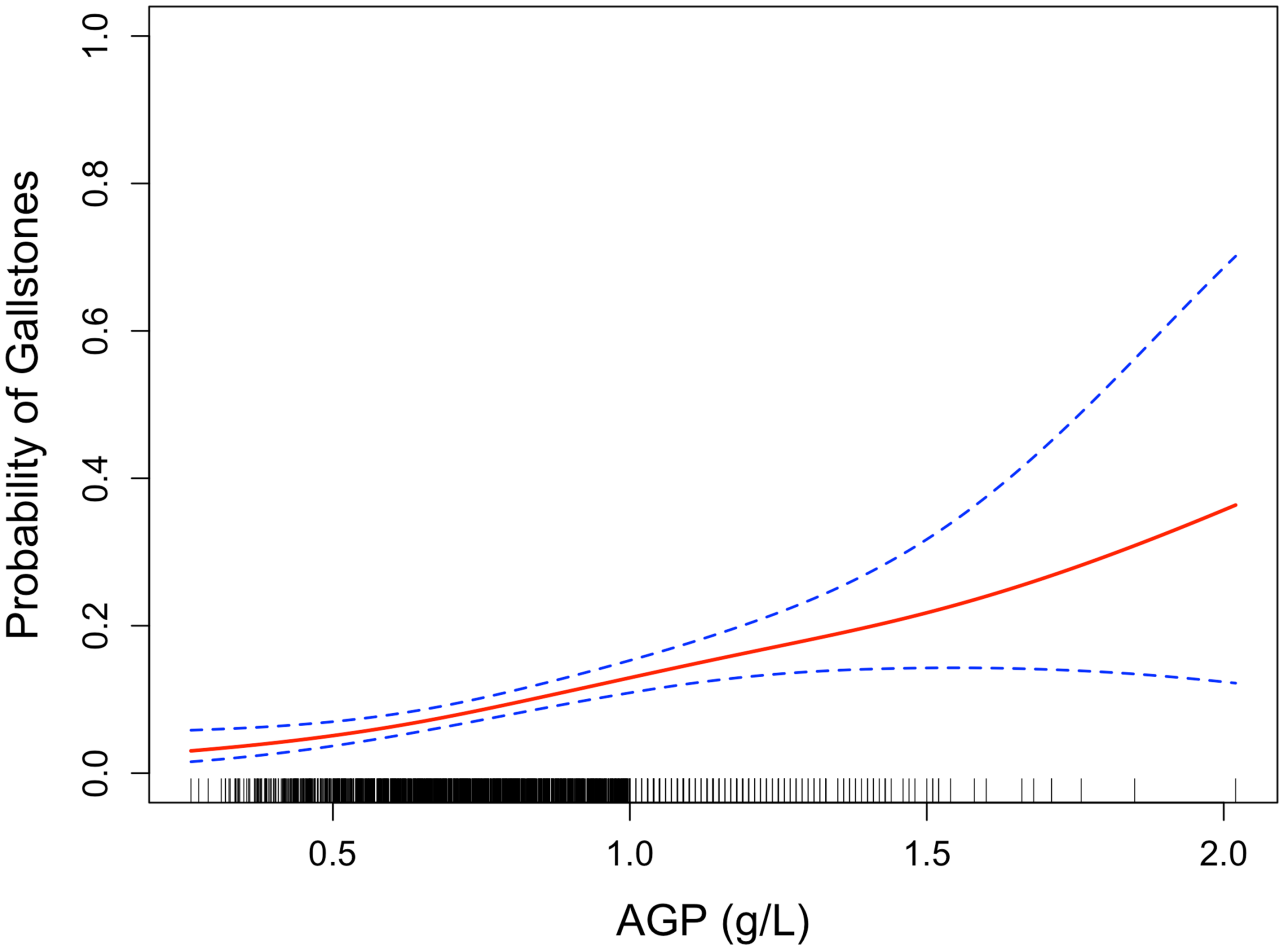
Smoothed curve analysis between AGP and gallstone risk by GAM. The red solid line represents the relationship between AGP and gallstone risk, while the dashed line indicates the 95% confidence interval (CI).

Smoothed curve fitting by the GAM displayed positive relationship between AGP and gallstone risk (Figure 2).

### 3.3 Subgroup analyses

Subgroup analysis revealed a positive association between higher levels of AGP and the risk of gallstone in several clinical categories (Table 3). Significant associations were observed in older adults aged 40–59 years, Non-Hispanic White individuals. Additionally, those with college or above, ratio of family income to poverty >3.5, no hypertension, no diabetes, alcohol consumers and smokers showed a significant positive correlation between higher AGP and gallstone risk (*p* < 0.05).

**Table 3:** Subgroup analyses for the association between AGP and gallstone risk.

Subgroups	Tertiles of AGP			*p for interaction*
	T1	T2	T3	
	Ref	OR (95%CI) *p*	OR (95%CI) *p*	
Age (years)				0.704
20–39	1.00	0.98 (0.48, 2.00) 0.954	1.11 (0.52, 2.38) 0.788	
40–59	1.00	2.23 (0.99, 5.04) 0.068	**3.17 (1.28, 7.86) 0.022**	
Race/Ethnicity				0.053
Non-Hispanic White	1.00	1.52 (0.47, 4.92) 0.494	**3.10 (1.26, 7.66) 0.025**	
Non-Hispanic Black	1.00	1.60 (0.27, 9.63) 0.617	0.52 (0.08, 3.38) 0.513	
Other	1.00	1.22 (0.64, 2.32) 0.555	0.75 (0.33, 1.71) 0.508	
Education				0.685
Less than high school	1.00	0.68 (0.12, 3.84) 0.670	0.62 (0.16, 2.42) 0.505	
High school	1.00	0.49 (0.15, 1.63) 0.265	0.44 (0.10, 1.89) 0.289	
College or above	1.00	1.92 (0.90, 4.11) 0.108	**2.45 (1.41, 4.26) 0.005**	
Ratio of family income to poverty				**0.011**
<1.3	1.00	0.34 (0.12, 0.95) 0.053	0.53 (0.22, 1.25) 0.163	
1.3–3.5	1.00	2.95 (0.85, 10.21) 0.103	3.07 (0.74, 12.82) 0.139	
>3.5	1.00	1.84 (0.67, 5.03) 0.251	**3.54 (1.42, 8.83) 0.013**	
Hypertension				0.600
Yes	1.00	0.74 (0.27, 2.05) 0.573	0.60 (0.18, 1.95) 0.408	
No	1.00	1.61 (0.79, 3.27) 0.206	**2.32 (1.22, 4.40) 0.019**	
Diabetes				0.388
Yes	1.00	2.78 (0.44, 17.42) 0.300	2.24 (0.46, 10.99) 0.344	
No	1.00	1.41 (0.77, 2.57) 0.279	**1.98 (1.14, 3.42) 0.025**	
Obesity				0.397
Yes	1.00	1.88 (0.84, 4.21) 0.143	2.48 (1.00, 6.17) 0.065	
No	1.00	0.82 (0.38, 1.76) 0.613	1.06 (0.53, 2.15) 0.869	
Alcohol intake				0.742
Yes	1.00	1.57 (0.62, 4.02) 0.354	**2.26 (1.27, 4.04) 0.012**	
No	1.00	1.34 (0.62, 2.91) 0.466	1.28 (0.44, 3.76) 0.653	
Smoking				0.103
Yes	1.00	1.69 (0.58, 4.98) 0.351	**3.31 (1.20, 9.09) 0.032**	
No	1.00	1.43 (0.78, 2.61) 0.262	1.23 (0.60, 2.52) 0.582	

Results were adjusted for age, race/ethnicity, education, ratio of family income to poverty, alcohol intake, obesity, hypertension, diabetes, smoking, sedentary activity, total cholesterol, hs-CRP, total energy intake, total fiber intake, total fat intake, total cholesterol intake, total moisture intake, except for the variable itself. All analyses were adjusted for the survey weights of the National Health and Nutrition Examination Survey (NHANES). AGP, alpha-1 acid glycoprotein; hs-CRP, high-sensitivity C-reactive protein; OR, odds ratio; CI, confidence interval. The bolded values represent subgroups with statistical significance.

Potential subgroup interactions were assessed using the likelihood ratio test. Among all subgroups, only the ratio of family income to poverty showed a significant interaction (*p for interaction* < 0.05). No other significant interactions were observed across the subgroups. Multiple comparisons were not adjusted, as these analyses were exploratory and hypothesis-generating in nature.

### 3.4 Sensitivity analysis

A sensitivity analysis was conducted on the multiply imputed data to assess the robustness of the findings. The analysis divided the data into weighted tertiles based on AGP levels: T1 [0.261–0.672], T2 [0.672–0.880], and T3 [0.880–2.760]. The results demonstrated that the estimates derived from the imputed data were consistent with those obtained from the original data (Supplementary Tables 3, 4).

## 4 Discussion

This study analyzed data from the NHANES 2017–2020 and 2021–2023 cycles, focusing on the association between AGP levels and the risk of gallstones in a total of 1,903 female adult participants. Weighted logistic regression revealed a significant positive association between AGP levels and gallstone risk, even after adjusting for all covariates (OR: 3.07; 95% CI: 1.16–8.11; *p* = 0.036). Specifically, after full adjustment, compared to the first tertile (T1), participants in the third tertile (T3) showed a statistically significant elevated risk (OR: 1.87; 95% CI: 1.11–3.14; *p* = 0.030). Smoothed curve analysis revealed positive relationship between AGP and gallstone risk. Subgroup analyses further supported the positive association between higher AGP levels and gallstone risk across various demographic and clinical categories. Sensitivity analyses, including MI, confirmed the robustness of these findings.

Previous studies have suggested a positive correlation between AGP levels in bile and serum with gallstones, indicating that AGP in bile may influence the nucleation time of cholesterol stones (14, 21). This study analyzed a larger and more representative sample from NHANES, specifically focusing on the association between AGP and gallstones in adult women. The results are consistent with previous findings, further supporting the role of AGP in gallstone formation.

As an inflammation-associated acute-phase protein, AGP may play a significant role in gallstone formation through several plausible biological mechanisms. Inflammatory processes are well-established contributors to gallstone pathogenesis, particularly within the context of metabolic syndrome, where chronic low-grade inflammation is a hallmark feature. Elevated AGP levels, which reflect systemic inflammation, could contribute to an immune environment that facilitates gallstone formation. Firstly, AGP is closely associated with cytokines such as IL-6 and TNF-α, which are critical mediators of chronic systemic inflammation (22). These cytokines are known to impair gallbladder motility by disrupting smooth muscle function, leading to bile stasis, which is a key risk factor for gallstone formation (23). Elevated AGP levels may serve as both a marker and a contributor to this inflammatory state, linking systemic inflammation to gallstone pathogenesis. Secondly, AGP may influence bile composition and metabolism, which are central to gallstone pathogenesis. Cholesterol supersaturation of bile is a primary driver of gallstone formation, and chronic inflammation disrupts hepatic bile acid regulation. AGP may interfere with nuclear receptor pathways, particularly farnesoid X receptor (FXR) (13), a key regulator of bile acid synthesis via CYP7A1. Impaired FXR signaling, potentially exacerbated by elevated AGP levels, could result in decreased bile acid secretion and increased cholesterol saturation, fostering a bile environment conducive to cholesterol crystallization and gallstone formation. Finally, metabolic syndrome provides a relevant framework to understand the role of AGP in gallstone formation. Elevated AGP levels in metabolic syndrome likely reflect a state of chronic systemic inflammation driven by central obesity, insulin resistance, and dyslipidemia. Insulin resistance is particularly relevant, as it impairs hepatic bile acid synthesis and increases biliary cholesterol secretion (24, 25). Lee et al. (26) found that excessive bile acids strongly induce AGP, which inhibits adipocyte differentiation and significantly suppresses the gene expression of adipogenic transcription factors, such as C/EBPβ, KLF5, C/EBPα, and PPARγ. While this response may confer anti-obesity effects, it may also alter lipid metabolism in ways that predispose individuals to cholesterol supersaturation of bile. Thus, AGP may act as a mediator by linking metabolic dysfunction, chronic inflammation, and bile dysregulation, creating conditions favorable for gallstone formation.

Clinically, these findings highlight AGP as a potential biomarker for assessing gallstone risk, especially in women with other known risk factors. Our findings, after adjusting for hs-CRP, suggest that AGP could serve as an independent marker of gallstone risk beyond the general inflammatory markers currently used in clinical practice. However, further studies are needed to evaluate its predictive value and clinical utility, particularly in identifying individuals at higher risk who might benefit from targeted interventions.

### 4.1 Strengths of this study

Firstly, utilizing data from NHANES 2017–2020 and 2021–2023, this study benefits from its national representativeness, making the findings generalizable to the broader U.S. adult population. Secondly, the study thoroughly adjusted for key demographic and lifestyle confounders, enhancing the reliability of the observed association between AGP and gallstone risk. Additionally, the study employed robust statistical methods, including weighted logistic regression and MI to validate the findings. Finally, this study systematically explores the link between AGP and gallstone risk in women, contributing to the understanding of this association and suggesting new avenues for future research.

### 4.2 Limitations

Firstly, the cross-sectional design limits causal inference, making it unclear whether elevated AGP levels contribute to gallstone formation or are a consequence of the disease. Secondly, gallstone disease was defined based on self-reported interview data rather than medical imaging or clinical diagnoses, which may introduce recall bias or misclassification. This limitation is inherent in large-scale population surveys like NHANES, and future studies should aim to use objective diagnostic measures, such as abdominal ultrasound, to confirm gallstone presence. Thirdly, while we adjusted for several key demographic and lifestyle confounders, other potential risk factors, such as fasting status and a history of biliary tract disease, were not comprehensively accounted for due to limitations in the dataset. These factors have been reported as important contributors to gallbladder-related conditions (27) and may influence the observed association. Finally, the study’s focus on American women aged 20–49 restricts generalizability to men and other age groups. Expanding the study population in future research would provide a more comprehensive understanding of AGP’s association with gallstone disease. Despite these limitations, identifying interventions to reduce AGP levels could have significant clinical implications, potentially aiding in gallstone prevention and management.

### 4.3 Future directions

Future longitudinal studies are needed to clarify causality and investigate AGP’s biological role in gallstone formation. Specifically, prospective cohort studies incorporating objective outcome measures, such as imaging techniques (e.g., abdominal ultrasound), are essential to confirm the presence of gallstones. The inclusion of ultrasound data would address potential misclassification bias due to self-reported gallstone diagnoses. Furthermore, future studies should incorporate a broader panel of inflammatory and metabolic markers, such as interleukins, tumor necrosis factor-alpha (TNF-α), adiponectin, and lipid profiles, to disentangle the causal pathways underlying the AGP-gallstone association. These markers would provide a more comprehensive understanding of the interplay between systemic inflammation, bile metabolism, and gallstone pathogenesis. Finally, mechanistic studies using animal models or human bile samples could elucidate AGP’s specific role in bile supersaturation, mucin secretion, and cholesterol crystallization. Identifying interventions to reduce AGP levels could have significant clinical implications, particularly for individuals at high risk for gallstone formation. Such interventions might include lifestyle modifications, anti-inflammatory therapies, or metabolic-targeted treatments, which warrant further exploration.

## 5 Conclusion

AGP is significantly and positively associated with gallstone risk in U.S. female adults. These findings suggest that AGP may play a role in gallstone risk, highlighting the need for further research to explore the underlying mechanisms and to investigate these associations in different populations and settings.

## Data Availability

Publicly available datasets were analyzed in this study. This data can be found here: https://www.cdc.gov/nchs/nhanes/index.htm.

## Footnotes

^1^ https://www.cdc.gov/nchs/nhanes/index.htm
