# Supplementary material for: Serum alpha-1 acid glycoprotein and gallstone risk in US adult women: a cross-sectional analysis of the NHANES
Source: Front Nutr. 2025 Feb 12;12:1527717. doi: 10.3389/fnut.2025.1527717 (PMC11860086; doi:10.3389/fnut.2025.1527717)
Supplement: Supplementary file 1 [file Table_1.docx]

Supplementary Material

**Supplementary Table 1.** Covariates missingness rates.

| **Covariates** | **Missing rate** |
| --- | --- |
| **Race/Ethnicity** | 0 |
| **Education** | 0.07% |
| **Age**, years | 0 |
| **Ratio of family income to poverty** | 11.48% |
| **Alcohol intake** | 11.22% |
| **Diabetes** | 1.44% |
| **Hypertension** | 0 |
| **Smoking** | 0.07% |
| **Obesity** | 1.23% |
| **Sedentary activity**, minutes | 0.47% |
| **Total cholesterol**, mmol/L | 1.05% |
| **Total energy intake**, kcal | 15.95% |
| **Total fiber intake**, g | 15.95% |
| **Total fat intake**, g | 15.95% |
| **Total cholesterol intake**, g | 15.95% |
| **Total moisture intake**, g | 15.95% |
| **High sensitivity C-reactive protein**, mg/L | 0.72% |

**Supplementary Table 2.** GVIFs adjusted for degrees of freedom in the complete model (Model 3).

| **Variables** | **GVIFs** |
| --- | --- |
| **Race/Ethnicity** | 1.86 |
| **Education** | 2.11 |
| **Age**, years | 2.47 |
| **Ratio of family income to poverty** | 2.64 |
| **Alcohol intake** | 2.43 |
| **Diabetes** | 2.07 |
| **Hypertension** | 1.93 |
| **Smoking** | 2.44 |
| **Obesity** | 2.02 |
| **Sedentary activity**, minutes | 2.21 |
| **Total cholesterol**, mmol/L | 2.12 |
| **Total energy intake**, kcal | 4.36 |
| **Total fiber intake**, g | 2.66 |
| **Total fat intake**, g | 4.93 |
| **Total cholesterol intake**, g | 2.27 |
| **Total moisture intake**, g | 2.20 |
| **AGP tertiles** | 1.79 |
| **High sensitivity C-reactive protein**, mg/L | 2.10 |

**Abbreviations:** AGP, alpha-1 acid glycoprotein. GVIFs, generalized variance inflation factors.

**Supplementary Table 3.** Distributions of variables in original cases compared to multiple imputation.

|  | **Original** | **Overall_imp1** | **Overall_imp2** | **Overall_imp3** | **Overall_imp4** | **Overall_imp5** |
| --- | --- | --- | --- | --- | --- | --- |
|  | 1903 | 2771 | 2771 | 2771 | 2771 | 2771 |
| **Race/Ethnicity**, n (%) |  |  |  |  |  |  |
| Non-Hispanic White | 804 (58.2) | 1105 (39.9) | 1105 (39.9) | 1105 (39.9) | 1105 (39.9) | 1105 (39.9) |
| Non-Hispanic Black | 384 (12.8) | 548 (19.8) | 548 (19.8) | 548 (19.8) | 548 (19.8) | 548 (19.8) |
| Other | 715 (28.9) | 1118 (40.3) | 1118 (40.3) | 1118 (40.3) | 1118 (40.3) | 1118 (40.3) |
| **Education**, n (%) |  |  |  |  |  |  |
| Less than high school | 215 (7.7) | 351 (12.7) | 351 (12.7) | 351 (12.7) | 351 (12.7) | 351 (12.7) |
| High school | 331 (19.9) | 503 (18.2) | 503 (18.2) | 503 (18.2) | 504 (18.2) | 504 (18.2) |
| College or above | 1357 (72.5) | 1917 (69.2) | 1917 (69.2) | 1917 (69.2) | 1916 (69.1) | 1916 (69.1) |
| **Age**, years |  |  |  |  |  |  |
| 20-39 | 1242 (67.3) | 1800 (65.0) | 1800 (65.0) | 1800 (65.0) | 1800 (65.0) | 1800 (65.0) |
| 40-49 | 661 (32.7) | 971 (35.0) | 971 (35.0) | 971 (35.0) | 971 (35.0) | 971 (35.0) |
| **Ratio of family income to poverty**, n (%) |  |  |  |  |  |  |
| <1.3 | 586 (23.3) | 875 (31.6) | 891 (32.2) | 874 (31.5) | 892 (32.2) | 875 (31.6) |
| 1.3–3.5 | 690 (35.6) | 1041 (37.6) | 1036 (37.4) | 1050 (37.9) | 1030 (37.2) | 1042 (37.6) |
| >3.5 | 627 (41.1) | 855 (30.9) | 844 (30.5) | 847 (30.6) | 849 (30.6) | 854 (30.8) |
| **Alcohol intake**, n (%) |  |  |  |  |  |  |
| No | 821 (38.5) | 1189 (42.9) | 1201 (43.3) | 1198 (43.2) | 1199 (43.3) | 1210 (43.7) |
| Yes | 1082 (61.5) | 1582 (57.1) | 1570 (56.7) | 1573 (56.8) | 1572 (56.7) | 1561 (56.3) |
| **Diabetes**, n (%) |  |  |  |  |  |  |
| No | 1738 (92.8) | 2536 (91.5) | 2541 (91.7) | 2537 (91.6) | 2540 (91.7) | 2536 (91.5) |
| Yes | 165 (7.2) | 235 ( 8.5) | 230 ( 8.3) | 234 ( 8.4) | 231 ( 8.3) | 235 ( 8.5) |
| **Hypertension**, n (%) |  |  |  |  |  |  |
| No | 1542 (83.9) | 2238 (80.8) | 2238 (80.8) | 2238 (80.8) | 2238 (80.8) | 2238 (80.8) |
| Yes | 361 (16.1) | 533 (19.2) | 533 (19.2) | 533 (19.2) | 533 (19.2) | 533 (19.2) |
| **Obesity**, n (%) |  |  |  |  |  |  |
| No | 1057 (58.9) | 1572 (56.7) | 1576 (56.9) | 1570 (56.7) | 1579 (57.0) | 1572 (56.7) |
| Yes | 846 (41.1) | 1199 (43.3) | 1195 (43.1) | 1201 (43.3) | 1192 (43.0) | 1199 (43.3) |
| **Smoking**, n (%) |  |  |  |  |  |  |
| No | 1304 (68.9) | 1922 (69.4) | 1923 (69.4) | 1923 (69.4) | 1923 (69.4) | 1922 (69.4) |
| Yes | 599 (31.1) | 849 (30.6) | 848 (30.6) | 848 (30.6) | 848 (30.6) | 849 (30.6) |
| **Sedentary activity**, minutes | 360.59 (206.01) | 344.96 (210.34) | 345.05 (210.44) | 345.56 (210.46) | 346.19 (210.46) | 345.12 (210.40) |
| **Total energy intake**, kcal | 1888.72 (798.16) | 1892.93 (801.53) | 1895.25 (809.59) | 1910.77 (821.73) | 1906.83 (823.92) | 1896.46 (815.05) |
| **Total fiber intake**, g | 14.82 (10.14) | 15.03 (9.73) | 14.98 (9.68) | 15.18 (9.77) | 15.03 (9.77) | 15.14 (9.86) |
| **Total fat intake**, g | 79.52 (40.98) | 78.30 (41.39) | 78.75 (41.76) | 79.38 (42.51) | 79.72 (42.90) | 79.13 (42.38) |
| **Total cholesterol intake**, g | 275.48 (223.16) | 279.98 (230.93) | 278.87 (232.83) | 283.89 (236.27) | 286.22 (236.19) | 280.62 (232.32) |
| **Total moisture intake**, g | 2843.58 (1320.13) | 2816.66 (1350.24) | 2801.14 (1342.61) | 2808.97 (1352.02) | 2773.32 (1354.99) | 2818.56 (1362.64) |
| **Total cholesterol**, mmol/L | 4.72 (0.90) | 4.69 (0.91) | 4.69 (0.91) | 4.69 (0.91) | 4.69 (0.91) | 4.69 (0.91) |
| **High sensitivity C-reactive protein**, mg/L | 4.30 (7.03) | 4.57 (7.87) | 4.57 (7.86) | 4.58 (7.87) | 4.57 (7.86) | 4.57 (7.87) |
| **AGP**, g/L | 0.79 (0.23) | 0.80 (0.24) | 0.80 (0.24) | 0.80 (0.24) | 0.80 (0.24) | 0.80 (0.24) |
| **Gallstones**, n (%) |  |  |  |  |  |  |
| No | 1718 (90.1) | 2501 (90.3) | 2501 (90.3) | 2501 (90.3) | 2501 (90.3) | 2501 (90.3) |
| Yes | 185 (9.9) | 270 ( 9.7) | 270 ( 9.7) | 270 ( 9.7) | 270 ( 9.7) | 270 ( 9.7) |

Continuous variables are presented as means with standard deviations. The data was **not adjusted** for the survey weights of the National Health and Nutrition Examination Survey (NHANES).

**Abbreviations:** AGP, alpha-1 acid glycoprotein.

**Supplementary Table 4.** Weighted multivariate regression analysis results with multiple imputation data.

| **Exposure** | **Model 1** | | **Model 2** | | **Model 3** | |
| --- | --- | --- | --- | --- | --- | --- |
|  | **OR (95% CI)** | ***p*** | **OR (95% CI)** | ***p*** | **OR (95% CI)** | ***p*** |
| **AGP (continuous)** | 6.69 (3.64, 12.30) | < 0.001 | 6.01 (3.37, 10.71) | < 0.001 | 2.42 (0.99, 5.91) | 0.052 |
| **Tertiles of AGP** |  |  |  |  |  |  |
| T1, [0.261–0.672] | Ref. |  | Ref. |  | Ref. |  |
| T2, [0.672–0.880] | 2.21 (1.38, 3.54) | 0.002 | 2.12 (1.31, 3.43) | 0.004 | 1.47 (0.91, 2.38) | 0.122 |
| T3, [0.880–2.760] | 3.69 (2.42, 5.64) | < 0.001 | 3.39 (2.28, 5.05) | < 0.001 | 1.70 (1.07, 2.69) | 0.035 |

Model 1: Unadjusted.

Model 2: Adjusted for age, race/ethnicity, education, ratio of family income to poverty.

Model 3: Adjusted for age, race/ethnicity, education, ratio of family income to poverty, alcohol intake, obesity, hypertension, diabetes, smoking, sedentary activity, total cholesterol, high sensitivity C-reactive protein, total energy intake, total fiber intake, total fat intake, total cholesterol intake, total moisture intake.

All results were adjusted for the survey weights of the National Health and Nutrition Examination Survey (NHANES).

**Abbreviations:** AGP, alpha-1 acid glycoprotein; OR, odds ratio; CI, confidence interval.
